# Alignment Between Cardiologists and AI-Driven Diagnostic Systems: Mixed Methods Study

**DOI:** 10.2196/83541

**Published:** 2026-05-20

**Authors:** Mahdi Mahdavi, Sarah White, Sandeep S Hothi, Chris Flood, Rosica Panayotova, Daniel Frings

**Affiliations:** 1 Department of Management and Enterprise, Faculty of Business and Law, University of Roehampton, London, United Kingdom; 2 Bernard Lown Scholars in Cardiovascular Health Programme, Harvard T.H. Chan School of Public Health, Boston, MA, United States; 3 School of Health and Medical Sciences, City St George's, University of London, London, United Kingdom; 4 Department of Cardiology, Royal Wolverhampton NHS Trust, Wolverhampton, United Kingdom; 5 College of Medical and Dental Sciences, Institute of Cardiovascular Sciences, University of Birmingham, Birmingham, United Kingdom; 6 School of Nursing and Midwifery, London South Bank University, London, United Kingdom; 7 Central London Community Healthcare NHS TrustLondon, United Kingdom; 8 Stockport NHS Foundation Trust, Stockport, United Kingdom; 9 College of Health and Life Sciences, London South Bank University, 103 Borough Road, London, SE1 0AA, United Kingdom, 44 20 7815 7815

**Keywords:** cardiac imaging, echocardiography, stress, clinical reasoning, artificial intelligence, cardiology

## Abstract

**Background:**

The clinical value of artificial intelligence (AI)–based diagnostic systems depends not only on their accuracy but also on how well their outputs integrate with clinicians’ judgments in practice. Critical knowledge gaps remain regarding diagnostic concordance between AI and clinicians in stress echocardiography interpretation, patient characteristics predicting discordance, and how cardiologists respond when AI recommendations conflict with their clinical diagnoses.

**Objective:**

This study examined the diagnostic alignment between an AI-driven stress echocardiography system (EchoGo Pro [EGP]) and cardiologists’ diagnoses of coronary artery disease (CAD), identified predictors of concordance and AI scan rejection, and explored cardiologists’ decision-making strategies when disagreements arise.

**Methods:**

We conducted mixed methods research. The quantitative study analyzed concordance between EGP and cardiologists using data from 854 participants with suspected CAD in the multicenter PROTEUS randomized controlled trial. Logistic regression identified predictors of agreement, disagreement, and scan rejection, adjusting for age, sex, smoking status, BMI, and cardiovascular risk factors (hypertension, hypercholesterolemia, diabetes, family history of CAD, and prior CAD events). To gain deeper insight into discordance, we conducted a qualitative study analyzing survey responses from 61 UK consultant cardiologists recruited via Qualtrics, exploring their perceptions of AI tools, the risks of following discordant AI recommendations, and their typical responses to AI-clinician disagreement.

**Results:**

EGP and cardiologists agreed in 60% (512/854) of the cases, but agreement was significantly lower among patients with hypertension (OR 0.58, 95% CI 0.38‐0.89; *P*=.01), diabetes (OR 0.56, 95% CI 0.35‐0.90; *P*=.02), and pre-existing CAD (OR 0.48, 95% CI 0.30‐0.77; *P*=.002). EGP rejected 26.1% (222/854) of the scans due to insufficient image quality, with rejection significantly more common in male patients (*β*=0.35; *P*=.03) and those with a family history of CAD. If a positive CAD diagnosis was assigned when either cardiologists or EGP identified CAD, the proportion of positive cases increased from 17.9% (153/854) to 22.1% (189/854), potentially identifying additional at-risk patients. Survey respondents (50/60, 85% male; 26/57, 46% aged 40-49 years; 39/61, 64% White) required 65% to 69% confidence in their initial diagnosis to justify disregarding contradictory AI recommendations. The survey findings revealed cardiologists treated AI recommendations as advisory rather than definitive. When facing discordance, they retained confidence in their judgment and sought corroboration through additional testing, data review, or second opinions rather than deferring to AI. Paradoxically, cardiologists with higher confidence in AI tools required greater confidence in their own diagnosis to disregard AI recommendations (*β*=7.73; *P*=.02). Cardiologists attributed discordance primarily to AI’s inability to incorporate patient history, comorbidities, and broader clinical context.

**Conclusions:**

EGP shows promise as an adjunctive tool but struggles with multimorbid patients and exhibits high, uneven rejection rates. Cardiologists use AI to prompt scrutiny, not replace judgment. Future systems need to integrate wider patient data with imaging and minimize bias through representative training to avoid exacerbating inequities.

## Introduction

The alignment between artificial intelligence (AI) assessments and human interpretations of health conditions influences clinicians’ confidence in diagnostic accuracy and the perceived use of AI systems in practice [1-3]. AI-driven tools have shown considerable promise in image processing in health care [4,5]. However, the clinical value of these tools depends not only on their accuracy against a reference standard but also on how their outputs integrate with clinicians’ judgments [6].

Studies on AI applied to health care suggest that clinicians are generally optimistic about AI but remain cautious, emphasizing transparency, bias, and responsibility, and often describe AI as an advisory rather than authoritative tool [6,7]. When AI and clinician judgments converge, this alignment can support timely and cost-effective care by reinforcing decision-makers’ confidence and potentially reducing the need for further testing [8]. However, when discordance arises, the extent to which clinicians rely on AI versus their own judgment and the factors driving such discrepancies are less well understood [7]. Therefore, understanding how AI outputs are interpreted in cases of discordance, the risks associated with following AI recommendations, and the conditions under which clinicians rely on AI rather than their own judgment is essential to fully harness the benefits of AI systems.

Stress echocardiography (SE) is a widely performed clinical investigation for the identification of coronary artery disease (CAD) [9]. It generates images of left ventricular wall motion acquired before and during exercise and/or pharmacological stress to make a clinical judgment on whether CAD is present, and which areas of the heart are affected. However, due to a degree of subjectivity in the test’s interpretation, clinical judgments may vary between clinicians [10]. Interobserver variability can lead to inconsistent classification of ischemia and variation in the patient journey, including referral for invasive angiography [11,12]. Additionally, SE is a resource-intensive test, requiring trained specialists to both perform and interpret the test. As a result, the demand for SE imaging is outpacing the capacity of cardiac specialists in many countries, particularly those with aging populations including the United Kingdom [13].

In SE tests, AI-based models have demonstrated high diagnostic accuracy for the detection and risk stratification of CAD, often achieving performance comparable to expert readers [6,10,14]. Their consistent and automated performance can enhance the accuracy of CAD diagnosis, while also reducing reporting time, providing a cost-effective second opinion, and minimizing interobserver variability [15,16].

EchoGo Pro (EGP), an AI-driven SE analysis system, has been developed for cardiac assessments and identifying potential ischemia [17]. EGP automatically analyzes SE images to estimate the probability of CAD and produces a structured binary risk report intended to support clinician decision-making. Initial validation studies have reported encouraging diagnostic accuracy and suggested that incorporating EGP into SE workflows may improve the detection of severe CAD compared with conventional interpretation alone [17].

Although systematic reviews consistently show that AI systems can achieve diagnostic performance comparable to health care professionals [3,6,18], most studies focus on accuracy metrics rather than on how clinicians respond in practice. Emerging evidence indicates that clinicians rarely respond to discordance in a simple accept-reject manner. Instead, they may ignore, selectively incorporate, negotiate, or conditionally defer to AI recommendations depending on their initial level of confidence in their own judgment, the perceived severity of the patient’s condition, and how clearly and convincingly the AI system explains its recommendation [19-21].

Yet, existing evidence is limited in scope and often based on noncardiology contexts, with little known about clinician behavior when AI outputs conflict with their own judgment, particularly in time-sensitive settings such as cardiac care [22]. Evidence is also scarce on which patient characteristics are associated with AI-clinician discordance, which factors predict AI rejection of scans, how cardiologists weigh AI outputs against their own judgment, and how perceived risk influences their willingness to follow AI recommendations [23,24]. Understanding how AI-clinician interaction unfolds in cardiac care, where rapid decisions can be lifesaving, is therefore critical for safe and effective implementation [24].

This mixed methods study integrates quantitative and qualitative analyses. The quantitative study uses clinical trial data to assess concordance between EGP and clinicians, examine patient characteristics associated with concordant and discordant cases, and identify demographic and clinical profiles linked to AI scan rejection or diagnostic divergence. The qualitative component explores cardiologists’ broader perceptions and experiences with AI tools, providing insight into attitudes, barriers, and contextual factors influencing adoption and trust. Together, these findings inform future development and implementation strategies for AI in cardiology. The analysis plan was preregistered on the Open Science Framework.

## Methods

### Overview of Research Design

We conducted a mixed methods study with 2 components, following a prespecified protocol published earlier [25]. The first was a secondary analysis of diagnostic agreement between EGP and cardiologists’ assessments of CAD in SE images. This analysis used data from 854 participants in the PROTEUS trial (for a broader evaluation of EGP, see below) [26]. The second component comprised a qualitative survey of consultant cardiologists, featuring open-ended questions designed to explore their perceptions and responses to disagreements with AI tools. Notably, this component considered AI tools in general rather than focusing specifically on EGP. Reporting of the agreement analysis adhered to the Guidelines for Reporting Reliability and Agreement Studies [27]. The survey component followed the STROBE (Strengthening the Reporting of Observational Studies in Epidemiology) checklist for cross-sectional studies [28].

### The Experimental Study

The PROTEUS trial was a randomized, multicenter, 2-armed study aimed at participants referred to SE clinics for the investigation of suspected CAD. The details of the PROTEUS research design are published by Woodward et al [26]. The trial randomized participants into a control group (SE assessment without EGP report) or an intervention group (SE assessment with EGP report). In the intervention group, SE images were analyzed by EGP, and a binary report produced by EGP indicating the probability of severe CAD was provided to the clinician alongside their own judgment [26]. Images failing quality criteria were flagged with a rejection report.

Cases were categorized as either concordant or discordant based on the alignment between EGP and clinician interpretation. Clinician interpretation was classified as positive for CAD if a stress-induced wall motion abnormality was observed, negative for CAD if no wall motion abnormality was induced, or inconclusive if the interpretation was abandoned or deemed indeterminate for inducible ischemia. The EGP risk rating classified cases as high risk of CAD (coronary stenosis ≥70%), low risk (coronary stenosis ≤70%), or uninterpretable if the image quality was insufficient for assessment.

Concordance was defined as agreement between the clinician and the EGP in either of the following scenarios: (1) a positive clinician interpretation and a high EGP risk rating or (2) a negative clinician interpretation and a low EGP risk rating. Discordance was defined as (1) a positive clinician interpretation paired with a low EGP risk rating or (2) a negative clinician interpretation paired with a high EGP risk rating.

The control variables consisted of age, sex, smoking status, BMI, and cardiovascular risk factors (CRFs). The data were collected through a combination of self-report questionnaires, physical measurements, and laboratory assessments. Age was categorized into the following groups: 18 to 39, 40 to 59, 60 to 80, and ≥81 years of age. Smoking status was self-reported and comprised current smokers, ex-smokers, or nonsmokers. BMI was calculated from measured height and weight, which were obtained during clinical examination. CRFs included hypertension, hypercholesterolemia, diabetes mellitus, positive family history of CAD, and history of CAD (including prior clinical events). Family history of CAD was self-reported, while other CRFs were determined through a combination of medical history review, physical measurements, and laboratory results. Specifically, hypertension was defined as measured blood pressure greater than or equal to 140/90 mm Hg or current use of antihypertensive medications [29]. Diabetes mellitus was diagnosed based on laboratory values (hemoglobin A_1c_ ≥6.5% [48 mmol/mol] or fasting blood sugar >126 mg/dL) or current use of antidiabetic medications [30]. Hypercholesterolemia and history of CAD were ascertained from medical records and confirmed through patient interview.

Descriptive statistics were used for demographics and clinical characteristics. Means and SDs were reported for normally distributed continuous variables, and frequencies and percentages were reported for categorical variables. We excluded cases where EGP reports were not used due to arriving late or being rejected (n=222, 26%). Logistic regression was used to identify the predictors of concordance or discordance and factors associated with AI image rejection. We used R for statistical analysis.

### The Survey

We collected survey responses from consultant cardiologists with expertise in SE (as a convenience sample) using a secure online survey platform and recruitment panel (Qualtrics). Demographic items were adapted from the UK Office for National Statistics framework [31], and screening questions ensured participant eligibility.

We included explanatory variables in the regression models to explore factors influencing cardiologists’ diagnostic confidence and their responses to AI recommendations. Years of experience as a cardiologist were grouped into 4 categories: 1 to 5, 6 to 10, 11 to 15, and 16 or more. The number of monthly SE reviews was categorized as follows: 0 to 20 tests, 21 to 50 tests, 51 to 100 tests, and more than 100 tests. Use of AI in the cardiac care pathway assessed exposure to AI in clinical practice. Participants were asked whether they used AI tools in the diagnosis or management of cardiac patients (yes or no). . Confidence in AI was measured using a 5-point Likert scale (1 = very low, 5 = very high). Respondents also indicated the level of confidence (on a scale from 1% to 100%) they would require in their initial diagnosis to disregard AI advice.

In addition, open-ended questions explored perceived risks of following AI in scenarios where AI tools contradicted clinicians’ judgment. Participants provided free-form text describing the potential risks of following the AI recommendation and further actions in response to two scenarios: (1) AI tools contradict the clinician’s diagnosis of CAD being present and (2) AI tools contradict the clinician’s diagnosis of CAD not being present. Of the 88 responses received, 61 met the inclusion criteria and were retained for analysis.

We used a mixed methods approach to analyze survey responses. Quantitative data were analyzed using regression models to examine associations between clinician characteristics (eg, years of experience, number of SE reviews per month, use of AI, and confidence in AI tools) and their reported confidence in diagnostic decision-making in hypothetical scenarios. In parallel, qualitative thematic analysis was conducted on open-text responses. This involved content analysis to identify common themes related to perceived risks of following AI recommendations and typical follow-up actions taken when AI tools and clinical assessments diverged.

The thematic analysis was based on the 6-phase framework of Braun and Clarke [32]: (1) familiarizing oneself with the data, (2) generating initial qualitative codes, (3) searching for themes, (4) reviewing themes, (5) defining and naming themes, and (6) producing a report. Two researchers independently coded responses and resolved discrepancies through discussion. Given the anonymous nature of the survey, member checking was not possible. However, qualitative themes were reviewed by 3 consultant cardiologists from PROTEUS field sites to enhance validity.

### Ethical Considerations

This study comprises 2 components: a clinical trial and a survey study. For the clinical trial, the protocol, informed consent form, participant information leaflet, and proposed advertising materials were reviewed by the Patient and Public Involvement and Engagement (PPIE) group and approved by the study sponsor and the North West—Preston Research Ethics Committee (REC) of the NHS Health Research Authority (reference: 21/NW/0199). The PROTEUS trial was registered on August 31, 2021, at ClinicalTrials.gov with registration number NCT05028179. For the survey study, ethical approval was obtained from the NHS Health Research Authority (IRAS number 315284) and the London South Bank University Ethics Panel (ETH2223-0164). All participants provided informed consent, and anonymized data were used for both study components. Survey participants were compensated for their participation via Qualtrics, with payments administered directly by the platform rather than the study team.

## Results

### The Experimental Study Results

#### Overview

Data from 854 participants in the intervention arm of the PROTEUS trial were used in the experimental analysis. Regional wall motion abnormalities were detected in 8% (71/893) of the participants, and 18% (156/893) received a clinical diagnosis of positive CAD (Table 1). Further details on participant demographics, lifestyle factors, and clinical history are available in Multimedia Appendix 1.

**Table 1.** Descriptive statistics and bivariate logistic regression results—primary outcome is concordance between clinician and EchoGo Pro (EGP) or divergence.

Patient characteristics	Convergent^a^ (n=512)	Divergent^b^ (n=113)	OR (95% CI)	*P* value
Sex, n				
Female	245	50	1	—^c^
Male	267	63	0.86 (0.57-1.30)	.49
Age group (y), n				
18‐39	20	3	1	—
40‐59	179	27	0.99 (0.28-3.57)	.99
60‐80	280	79	0.53 (0.15-1.84)	.32
>81	33	4	1.24 (0.25-6.11)	.79
Smoking status, n				
Current smoker	52	11	1	—
Ex-smoker	216	41	1.11 (0.54-2.32)	.77
Nonsmoker	240	60	0.85 (0.42-1.72)	.64
BMI, mean (SD)	29.0 (10.8)	29.8 (6.2)	0.99 (0.98-1.01)	.45
Control variables				
Has cardiovascular risk factors?, n	406	102	0.41 (0.21-0.80)	.008
Number of cardiovascular risk factors, mean (SD)	1.5 (1.2)	1.9 (1.0)	0.77 (0.65-0.92)	.003
Hypertension, n	254	71	0.58 (0.38-0.89)	.01
Hypercholesterolemia, n	214	58	0.68 (0.45-1.02)	.07
Diabetes, n	90	31	0.56 (0.35-0.90)	.02
Positive family history of coronary artery disease, n	182	41	0.97 (0.63-1.48)	.88
Previous CAD^d^ clinical events, n	44	14	0.66 (0.35-1.26	.21
Pre-existing coronary artery disease, n	85	33	0.48 (0.30-0.77)	.002

a Concordance refers to (1) both clinician and EchoGo Pro (EGP)–confirmed presence of coronary artery disease (CAD) or (2) both clinician- and EGP-confirmed absence of CAD.

b Discordance refers to (1) clinician-diagnosed CAD, but EGP did not confirm it, or (2) EGP-diagnosed CAD, but the clinician did not confirm it. The analysis focuses on data from the experimental study conducted specifically with the EGP tool.

c Not applicable.

d CAD: coronary artery disease.

#### Concordance and Discordance

Of the analyzed cases, 512 (60%) were classified as concordant (Table 1). Within this group, 38 (4.5%) cases demonstrated concordance on a positive CAD diagnosis, where both the clinician identified inducible ischemia and EGP reported a high risk of CAD. The remaining 474 (55.5%) cases were concordant on a negative diagnosis, with the clinician reporting no inducible ischemia and EGP indicating low risk.

A total of 113 (13.2%) cases were classified as discordant. Of these, 36 (4.2%) cases involved a clinician-assessed negative result for inducible ischemia, while EGP reported a high risk of CAD. Conversely, 77 (9%) cases involved a positive clinical interpretation for inducible ischemia, but EGP assessed the case as low risk.

If a positive diagnosis were assigned based on either a positive clinician interpretation or a high-risk EGP rating, the total number of positive CAD diagnoses would increase from 153 (17.9%) to 189 (22.1%).

EGP rejected 222 (26.1%) cases due to insufficient image quality, whereas clinicians classified only 10 (1.2%) cases as inconclusive. Concordance among rejected cases was very low, with only 3 (0.4%) cases showing agreement between EGP rejection and clinician inability to interpret.

#### Factors Predicting Concordance or Discordance

Table 2 presents the comparison of concordance and discordance rates across sex, age groups, and smoking status. Bivariate logistic regression analyses indicated no statistically significant differences in concordance or discordance between male and female participants, across age groups, or among different smoking statuses.

**Table 2.** Concordance and discordance between clinician interpretation and EchoGo Pro (EGP) report (experimental study).

	Artificial intelligence risk rating report^a^			
Clinician interpretation of presence or ischemia^b^	High, n (%)	Low, n (%)	Rejected, n (%)	Total, n (%)
Positive for inducible ischemia	38 (4.5)	77 (9)	38 (4.5)	153 (17.9)
Negative for inducible ischemia	36 (4.2)	474 (55.5)	181 (21.2)	691 (80.9)
Inconclusive or abandoned	0 (0)	7 (0.8)	3 (0.4)	10 (1.2)
Total	74 (8.7)	558 (65.3)	222 (26.1)	854 (100)

a Artificial intelligence risk rating report was classified as high risk of CAD (stenosis ≥70%) or low risk (stenosis <70%), or the stress echocardiography image was rejected due to poor quality.

b Clinician interpretation of the presence of ischemia was classified as a positive coronary artery disease if they diagnosed that wall motion abnormality was present, negative coronary artery disease if no wall motion abnormality was present, or the interpretation was inconclusive or abandoned for inducible ischemia. The analysis focuses on the data from the experimental study conducted specifically with the EchoGo Pro tool.

Having more CRFs or pre-existing CAD was associated with lower odds of concordance. Patients in concordant cases had fewer cardiovascular risk factors than those in discordant cases (mean 1.5, SD 1.2 vs mean 1.9, SD 1.0). Each additional cardiovascular risk factor was associated with lower odds of concordance between EGP and clinician interpretations (OR 0.77, 95% CI 0.65‐0.92; *P*=.003). The strongest associations with discordance were observed in patients with any cardiovascular risk factor (OR 0.41, 95% CI 0.21‐0.80; *P*=.008), pre-existing CAD (OR 0.48, 95% CI 0.30‐0.77; *P*=.002), and diabetes (OR 0.56, 95% CI 0.35‐0.90; *P*=.02). However, multivariate logistic regression suggested that no sociodemographic variable was associated with discordance or concordance.

#### EGP Rejecting Scans

We ran a multivariable logistic regression to examine whether sex, age group, smoking status, BMI, and cardiovascular risk factors were associated with the likelihood of SE image rejection by EGP (Table 3). Sex was the only demographic variable significantly associated with rejection: scans from male participants were more likely to be rejected than those from female participants (*β*=0.35, *P*=.03). Among clinical variables, hypercholesterolemia and family history of CAD showed significant associations with scan rejection. Individuals with hypercholesterolemia were less likely to have their scans rejected (*β*=−0.36; *P*=.04), whereas those with a positive family history of CAD were more likely to experience rejection (*β*=0.33; *P*=.049).

**Table 3.** Multivariate logistic regression results—primary outcome is artificial intelligence (AI) rejection of scan or conclusive scan^a^.

Patient characteristics	Coefficient (SE)	*P* value
Intercept	−1.28 (0.56)	.02
Age groups (y)		
18‐39	—^b^	—
40‐59	0.02 (0.46)	.97
60‐80	0.31 (0.45)	.50
>81	0.69 (0.53)	.19
Sex		
Female	—	—
Male	0.35 (0.17)	.03
Smoking		
Current smoker	—	—
Ex-smoker	−0.20 (0.28)	.48
Nonsmoker	−0.11 (0.27)	.68
BMI	0.00 (0.01)	.78
Cardiovascular risk factors		
Hypertension	0.03 (0.17)	.87
Hypercholesterolemia	−0.36 (0.17)	.04
Diabetes	−0.04 (0.21)	.85
Positive family history of coronary artery disease	0.33 (0.17)	.049
Previous CAD^c^ clinical events	0.20 (0.26)	.43

a The analysis focuses on the data from the experimental study conducted specifically with the EchoGo Pro tool.

b Not applicable.

c CAD: coronary artery disease.

### Survey Results

#### Overview

The data from 61 survey participants were included in the analysis. Most were male (50/60, 85%), aged 40 to 49 years (26/57, 46%), and White (39/61, 64%). Professional experience varied, with the majority having more than 5 years of experience as cardiologists (Multimedia Appendix 1). Over half of the survey participants reported using AI tools in cardiac care (33/61, 54%), including auto-indexing software (33/61, 54%) and decision-support software (23/61, 38%). The number of monthly SE tests reviewed ranged from 1 to over 100, with nearly half reviewing 21 to 50 tests per month.

#### Scenario Analysis

The scenario analysis used survey responses in which AI tools were considered in general rather than focusing on a specific system such as EGP. In scenario A (the cardiologist diagnosed CAD, but the AI tools contradicted this), the mean level of confidence in the initial diagnosis required to justify taking no further action was 69%. In scenario B (CAD was not identified by the cardiologist but was diagnosed by the AI tools), the corresponding mean confidence level was 65%.

Table 4 presents linear regression models examining how cardiologist characteristics relate to the confidence threshold required to disregard AI advice. Across both scenarios, this threshold was not significantly associated with years of experience as a cardiologist, the number of stress echocardiograms reviewed per month, or the current use of AI in the cardiac care pathway. However, in scenario A, higher confidence in AI tools was positively associated with the level of diagnostic confidence required to disregard an AI recommendation (*β*=7.73; *P*=.02). This suggests that cardiologists who are more trusting of AI systems are more cautious and require stronger conviction in their own clinical judgment before overriding AI-generated assessments.

**Table 4.** Regression models for scenario analysis: cardiologists’ confidence in their initial diagnosis^a^.

Predictor variable	Scenario A: cardiologist made a diagnosis of CAD^b^, but the AI^c^ tools contradicted^d^		Scenario B: CAD was not identified by cardiologists, but was diagnosed by AI tools^e^	
	Coefficient (SE)	*P* value	Coefficient (SE)	*P* value
Intercept	39.37 (12.07)	.002	55.49 (14.26)	<.001
Years of experience as a cardiologist				
1‐5 (reference)	—^f^	—	—	—
6‐10	−1.51 (7.39)	.84	0.45 (8.74)	.96
11‐15	10.21 (7.48)	.18	−1.21 (8.85)	.89
>16	0.90 (7.19)	.90	−0.52 (8.50)	.95
Number of monthly stress test reviews				
0‐20 tests (reference)	—	—	—	—
21‐50 tests	3.61 (6.83)	.60	−11.57 (8.07)	.16
51‐100 tests	4.13 (8.92)	.65	−8.72 (10.54)	.41
>101 tests	4.90 (7.83)	.53	−3.93 (9.26)	.67
AI in the cardiac care pathway				
No (reference)	—	—	—	—
Yes	−3.68 (5.06)	.47	2.88 (5.98)	.63
Confidence in AI tool^g^	7.73 (3.11)	.02	4.58 (3.67)	.22

a The analysis presented in this table is based on survey data where artificial intelligence tools are considered in general.

b CAD: coronary artery disease.

c AI: artificial intelligence.

d Confidence in initial diagnosis for not taking any further actions: 69%.

e Confidence in initial diagnosis for not taking any further actions: 65%.

f Not applicable.

g Confidence in AI was measured on a 5‑point Likert scale (1=very low, 5=very high). Confidence in their own diagnosis was measured on a scale from 1% to 100%. Regression coefficients are unstandardized.

#### Perceived Risk of Following AI Recommendations

In scenario A, the most perceived risk of following AI recommendations, considered broadly rather than specific to EGP, was the possibility of inaccurate diagnoses or inappropriate treatments, which could potentially cause harm. Other prominent concerns included AI limitations, such as lack of patient-specific context, inadequate training, and the early-stage development of AI tools, as well as legal liability, with cardiologists remaining ultimately responsible for patient care. Some participants, however, perceived minimal risk, viewing AI as a nonbinding decision-support tool.

In scenario B, the risk of inaccurate diagnosis emerged as a key theme in participants’ perceptions of the risks associated with following AI recommendations. Two additional prominent themes were the overuse of clinically ineffective but nonharmful services and the misuse of care—specifically, unnecessary treatments that could potentially harm patients or cause side effects. Participants expressed these concerns with statements such as: “Some treatments may not improve patient symptoms. Unnecessary intervention could be harmful,” and “Overtreatment, possible side effects of therapy, patient anxiety.”

#### Actions Taken Following AI Recommendations

Study participants were responsive to situations in which AI recommendations contradicted cardiologists’ clinical judgment regarding the presence or absence of CAD. Across both scenarios, the most frequently reported response was to initiate additional investigations. Another prominent theme was the pursuit of a second opinion, consultation, or advice, reflected in subthemes such as seeking a second opinion, requesting advice, or consulting peer colleagues. A third major theme involved reviewing broader patient data, including diagnostic tests and medical records, to verify or confirm information through a more comprehensive assessment. In a small number of cases, participants reported taking no further action. Finally, a less common theme was the repetition of tests or re-examination of existing information, suggesting an intent to audit or reassess the initial diagnosis and underlying data.

## Discussion

This mixed methods study combined PROTEUS trial data analysis with a cardiologist survey, examining how EGP’s stress echo assessments aligned with clinician interpretations and exploring attitudes toward AI-human discordance. EGP showed substantial overall agreement with clinicians but generated meaningful discordance, concentrated among patients with multiple cardiovascular risk factors and established CAD. Concordance was similar across sex, age, and smoking status but lower in patients with hypertension, diabetes, or pre-existing CAD. Over one-quarter of scans were rejected due to poor image quality, with rejection more common in male patients and those with a family history of CAD, and less frequent in patients with hypercholesterolemia.

Survey respondents described AI as supplementary rather than primary decision support. When facing discordance, cardiologists reported retaining confidence in their initial judgment and seeking corroboration through further investigations, clinical data review, or second opinions rather than deferring to AI. Those who expressed higher overall confidence in AI reported that they would require stronger confidence in their own diagnosis before disregarding an AI recommendation, particularly when the AI suggested disease they had not initially identified. Some expressed concerns that following discordant AI advice could trigger unnecessary investigations and overtreatment.

The concentration of discordance in patients with complex profiles highlights the limitations of image-only approaches [33]. This is clinically significant because patients with hypertension, diabetes, and pre-existing CAD face substantially higher risks of future events [34], yet accurate diagnosis in these critical groups is where EGP and clinicians most often diverged. EGP is trained on images [35], whereas clinicians integrate clinical history, comorbidities, and broader patient data. For example, patient-specific anatomical features can contribute to false positive findings by creating artifacts that mimic ischemia, highlighting how structural features influence diagnostic variability [36]. Consequently, future systems must move toward multimodal designs that incorporate anatomical features, clinical history, and comorbidities to better mirror diagnostic reasoning [23,36,37].

These findings highlight both EGP’s potential and its limitations. When used alongside expert interpretation, EGP can flag additional high-risk patients who might otherwise be missed. Given that missed CAD diagnoses cost approximately £4200 per patient versus £2300 for correct diagnoses [38], even modest gains in diagnostic yield may improve outcomes and reduce downstream costs. However, EGP alone often classified fewer positives than clinicians and failed to interpret rejected scans, reinforcing its current role as an adjunctive decision-support tool rather than a stand-alone solution.

We observed a nonsignificant trend toward increased false positives with EGP. At population scale, even minor increases affect service planning; a theoretical 1% rise in false positives across the United Kingdom’s 61,000 annual scans [35] would trigger approximately 610 unnecessary investigations, costing roughly £1.4 million. Beyond financial burdens, false positives generate patient anxiety and avoidable invasive procedures. Consequently, the trade-offs between enhanced sensitivity and the risks of over-diagnosis warrant careful consideration during AI upscaling.

A further challenge relates to EGP’s relatively high rate of scan rejection, which undermines its reliability and usability. Frequent noninterpretable outputs are likely to erode clinicians’ trust and limit the perceived usefulness of the system. Importantly, rejection was not uniform. Although mechanisms cannot be determined directly from our data, these differences are consistent with broader evidence that imbalances in training datasets, such as sex disparities or underrepresentation of certain clinical risk profiles, can lead to biased performance of AI models across demographic and clinical groups [39-41]. Addressing these inequities requires more representative training data, routine postdeployment monitoring across subgroups, and model refinement where necessary.

The survey findings clarify how such tools are likely to be used. Cardiologists framed AI as supportive rather than authoritative, responding to disagreements by seeking further tests, reviewing additional data, or obtaining second opinions, suggesting a tendency toward confirmation-oriented behavior rather than automation bias [22,42]. While this caution mitigates overreliance risks, frequent discordance may paradoxically increase downstream testing and costs. Clear guidance on managing AI-clinician disagreement and warranting additional investigations is therefore essential for implementation.

This study has several notable strengths. Leveraging data from the multicenter PROTEUS trial provided a robust and standardized framework for evaluating EGP across diverse clinical sites, reducing the influence of local practice patterns and supporting internal validity. The sizeable sample allowed the examination of agreement, disagreement, and scan rejection across clinically relevant subgroups. By explicitly modeling predictors of AI-clinician concordance and AI rejection, we begin to identify which patients may be better or worse served by current image-based systems. Finally, the mixed methods design offers a holistic understanding of both EGP’s technical behavior and how clinicians interpret and act on its recommendations in practice.

A few limitations should be borne in mind. First, the high scan rejection rate reduced the effective sample size for concordance analyses and may have introduced selection bias if rejected scans systematically differed from accepted scans in unmeasured ways. Second, although we included a range of demographic and clinical characteristics, other potentially important determinants of image quality and diagnostic decision-making, such as sonographer expertise, variation in acquisition protocols, and differences in operators’ familiarity with EGP, were unavailable but likely influence both AI performance and clinician interpretations [43,44]. Finally, both study components were conducted within a specific national health care context; EGP’s performance and acceptance may differ in other health systems or populations.

Beyond EGP, these findings have broader implications for AI implementation in cardiovascular imaging. Discordance concentrated in patients with hypertension, diabetes, and prior CAD reflects the limitations of image-only models that cannot incorporate symptoms, medications, and comorbidities as clinicians do. Future AI systems must integrate these clinical variables. The uneven rejection rates by sex and clinical profile require ongoing monitoring for bias after deployment. Additionally, cardiologists’ concerns about legal liability when AI recommendations conflict with their judgment must be addressed through clear clinical protocols and regulatory guidance. Further research is needed to establish evidence-based approaches for managing AI-clinician disagreement and ensuring equitable performance across patient populations.

In this mixed methods study, EGP agreed with cardiologists in most cases, but discordance clustered in patients with multiple cardiovascular risk factors or established coronary disease, and many scans were rejected, particularly in some patient groups. Cardiologists described using AI as supportive input rather than a decision-maker, typically responding to AI-clinician disagreement by ordering further tests, reviewing additional data, or seeking second opinions. Overall, these findings position EGP and similar tools as useful adjuncts that can highlight additional at-risk patients but cannot yet replace holistic clinical assessment. Future development should prioritize incorporating key clinical information alongside imaging, reducing and monitoring rejection and bias across subgroups, and embedding AI within clear clinical pathways so that disagreement is managed transparently and patient safety, efficiency, and equity are all improved.

## Supplementary material

10.2196/83541 Multimedia Appendix 1 Additional descriptive statistics and subgroup analyses of participants in the experimental study and cardiologist survey.

## Abbreviations

AI: artificial intelligence
CAD: coronary artery disease
CRF: cardiovascular risk factor
EGP: EchoGo Pro
SE: stress echocardiography
STROBE: Strengthening the Reporting of Observational Studies in Epidemiology
