# Supplementary material for: Alignment Between Cardiologists and AI-Driven Diagnostic Systems: Mixed Methods Study
Source: J Med Internet Res. 2026 May 20;28:e83541. doi: 10.2196/83541 (PMC13189529; doi:10.2196/83541)
Supplement: Multimedia Appendix 1 [file jmir-v28-e83541-s001.docx]

**Alignment between cardiologists and AI-driven diagnostic systems: Predictors of Agreement, Decisions, and Risks.**

Mahdi Mahdavi, Sarah White, Sandeep Hothi, Chris Flood, Rosica Panayotova, Daniel Frings

# Supplementary file 1: Additional results

Table 1. Descriptive statistics of sociodemographic variables and main outcomes

|  |  |  | Participants (n=893) | |
| --- | --- | --- | --- | --- |
|  |  |  | N | % |
| Socio-demographic variables | Sex | Male | 488 | 55 |
|  |  | Female | 405 | 45 |
|  | Age group | 18-39 | 30 | 3 |
|  |  | 40-59 | 285 | 32 |
|  |  | 60-80 | 520 | 58 |
|  |  | 81+ | 58 | 6 |
|  | Smoking status | Current smoker | 98 | 11 |
|  |  | Ex smoker | 358 | 40 |
|  |  | Non-smoker | 429 | 48 |
|  |  | Missing | 8 | 1 |
|  | Body Mass Index | | 28.6 (28.0-29.2)* | |
| Control variables | Cardiovascular risk factors | Yes | 730 | 82 |
|  |  | No | 163 | 18 |
|  | Cardiovascular risk factors | Hypertension | 464 | 29 |
|  |  | Hypercholesterolaemia | 371 | 23 |
|  |  | Diabetes | 170 | 11 |
|  |  | Positive Family History of coronary artery disease | 327 | 20 |
|  |  | Previous CAD clinical events | 86 | 5 |
|  |  | Other | 15 | 1 |
|  |  | None | 165 | 10 |
|  | Pre-existing CAD | Yes | 175 | 20 |
|  |  | No | 718 | 80 |
| Main outcomes | Regional wall motion abnormalities | Yes | 71 | 8 |
|  |  | No | 798 | 92 |
|  | Clinician interpretation of presence of CAD | Inconclusive or abandoned | 22 | 2 |
|  |  | Positive | 156 | 18 |
|  |  | Negative | 702 | 80 |
|  | Undergone invasive coronary angiography | Yes | 37 | 5 |
|  |  | No | 712 | 95 |
|  | Adjudication – trial cardiologist | No sig disease | 3 | 16 |
|  |  | Significant disease | 16 | 84 |

This refers to 95% confidence interval.

# The survey

Most participants of the survey were male (85%). The age of participants ranged from 30 to 70 years; 46% of participants were aged between 40-49 years. The largest ethnicity group was White comprising 64% of participants (see *Table 4* for other ethnicities). Most participants have worked as a cardiologist for longer than five years (*Table 4*). More than half of participants (54%) reported current use of AI in a cardiac care pathway (*Table 5*).

*Table 4. Demographic characteristics of the survey participants*

|  | Demographic information | Frequency | Percent (%) |
| --- | --- | --- | --- |
| Sex | Female | 8 | 14 |
|  | Male | 50 | 85 |
|  | Other | 2 | 3 |
| Age groups | 30-39 | 16 | 28 |
|  | 40-49 | 26 | 46 |
|  | 50-70 | 15 | 26 |
| Ethnicity | White | 39 | 64 |
|  | Asian/Asian British | 12 | 20 |
|  | Black/African/Caribbean/Black British | 3 | 5 |
|  | Mixed/Multiple Ethnic Groups | 2 | 3 |
|  | Other Ethnic Group | 5 | 8 |
| Years working as a cardiologist | 1-5 | 12 | 20 |
|  | 6-10 | 15 | 25 |
|  | 11-15 | 17 | 28 |
|  | 16+ | 17 | 28 |

*Table 5. Experience of cardiologists on the use of AI applications in cardiac care and reviewing stress echocardiography tests*

|  | AI in the cardiac care pathway | Frequency | Percent (%) |
| --- | --- | --- | --- |
| Currently using AI tools in cardiac care | Yes | 33 | 54 |
|  | No | 27 | 44 |
|  | Unknown | 1 | 2 |
| Currently using auto-indexing software in cardiac care | Yes | 33 | 54 |
|  | No | 28 | 46 |
| Currently using Decision Support Software in cardiac care | Yes | 23 | 38 |
|  | No | 38 | 62 |
| Number of monthly stress echo tests reviewed | 1-20 tests | 12 | 20 |
|  | 21-50 tests | 29 | 48 |
|  | 51-100 tests | 7 | 11 |
|  | 101+ tests | 13 | 21 |
